# Skin Microbiome in Health and Disease: Dysbiosis and the Emerging Role of Biotics in Therapy and Protection

**DOI:** 10.3390/ijms27156763

**Published:** 2026-07-28

**Authors:** Anna Łętocha, Małgorzata Miastkowska, Elżbieta Sikora

**Affiliations:** Department of Organic Chemistry and Technology, Faculty of Chemical Engineering and Technology, Cracow University of Technology, Warszawska 24, 31-155 Cracow, Poland; malgorzata.miastkowska@pk.edu.pl (M.M.); elzbieta.sikora@pk.edu.pl (E.S.)

**Keywords:** skin microbiome, probiotics, prebiotics, dysbiosis

## Abstract

The skin functions primarily as a protective physical shield, defending the body against harmful external factors such as invading microorganisms and toxic agents. When this barrier becomes impaired and skin microbiome homeostasis is disturbed, exacerbation of chronic skin diseases may occur, including atopic dermatitis, psoriasis, and acne. This issue became particularly important during the coronavirus pandemic, when it turned out that handwashing and disinfection removed not only impurities and pathogenic microorganisms but also beneficial components of the skin microbiome. This review provides a critical overview of recent advances in understanding the skin microbiome and its influence on skin health, as well as supportive therapies for the treatment of dermatoses. Contemporary therapeutic approaches focus not only on suppressing inflammatory symptoms but also on modulating the skin microbiome as part of a causal treatment strategy, in which prebiotics and probiotics play a significant role. Moreover, probiotics are also considered a valuable component of anti-aging approaches, as they may help counteract the detrimental effects of ultraviolet (UV) radiation on the skin. By reducing oxidative stress, strengthening the epidermal barrier, and exerting immunomodulatory effects, they may complement traditional photoprotection methods. Nevertheless, further research—particularly into their long-term efficacy and safety—is essential to support their broader clinical and consumer application.

## 1. Introduction

Human skin constitutes a complex ecosystem inhabited by numerous microorganisms, including bacteria, fungi, viruses, and mites. These microorganisms collectively form the skin microbiota [1]. Various skin structures—such as hair follicles, sebaceous and sweat glands, as well as the stratum corneum—serve as niches for distinct microbial communities. The richness of this microbiota was first systematically characterized in 2011 by Elizabeth Grice and Julia Segre [2]. Today, researchers emphasize the important role of the skin microbiome in maintaining proper skin function: protection against pathogenic microorganisms, strengthening of the immune system, and the ability to break down and produce various compounds [3,4,5,6,7]. The skin microbiota serves as the first line of defense against harmful environmental factors. It contributes to host immune defense, and one of its mechanisms involves inhibiting the growth of pathogens by producing bactericidal substances. The microbiota also participates in the development of adaptive immunity by modulating local cytokine production, as well as enhancing innate immunity by increasing the production of antimicrobial proteins [8].

Unfortunately, improper hygiene, cleansing, and daily skincare practices may alter the composition and function of the skin microbiota and disrupt skin microbiome homeostasis, leading to excessive skin dryness or skin disorders such as atopic dermatitis. For this reason, the impact of cosmetic formulations on the skin microbiome and the need to use microbiome-friendly raw materials, as well as to enrich formulations with pro- and prebiotic ingredients, have become not only a subject of scientific research [9,10] but also one of the leading trends in the cosmetics market.

This issue became particularly important during the coronavirus pandemic, when handwashing and disinfection were key elements of sanitary regulations. While hygienic and disinfectant products effectively cleanse the skin, removing impurities and pathogenic microorganisms, they also strip away beneficial components of the skin microbiome [11].

Probiotic and prebiotic substances have long been successfully used as ingredients in dietary supplements. Existing definitions of these terms were developed primarily in the context of nutritional applications. According to FAO/WHO, probiotics are “live microorganisms which, when administered in adequate amounts, confer a health benefit on the host” [12]. In cosmetics, however, these terms are used more broadly. Live microorganisms can only be used in cosmetic products to a very limited extent, as their viability and activity are difficult to control [11,13]. Limitations also arise from the strict microbiological purity requirements of cosmetic products [14].

To include live bacteria in cosmetic formulations, preservatives should be omitted. However, from the perspective of cosmetic safety, as well as the quality requirements imposed on cosmetic products (Regulation No. 1223/2009 on cosmetic products) [15], this is not possible—especially in the case of water-based formulations.

## 2. Skin Microbiome

The skin is the largest organ of the human body. It forms an ecosystem with a surface area of approximately 2 m^2^, but when considering its complexity (about 5 million skin appendages), the total area increases significantly to around 25 m^2^, making it the largest organ in the body [16,17,18].

**Figure 1:**
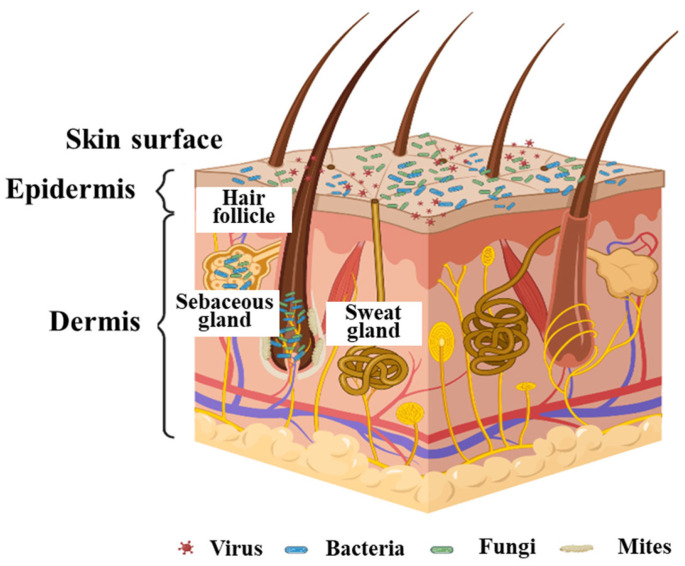
Diagram of the structure of the skin. Created in BioRender. Łętocha, A. (2026). https://BioRender.com/9lwx9lm (accessed on 3 July 2026).

The skin primarily acts as a protective physical barrier, safeguarding the body from harmful external agents such as invading microorganisms and toxic compounds. In addition, it serves as a boundary with the surrounding environment and is inhabited by a diverse array of microorganisms, including bacteria, fungi, and viruses, as well as mites. These organisms are typically grouped into resident, temporary, and transient populations [19,20]. Both microorganisms and mites occur not only on the surface of the skin but also deeper within structures such as hair follicles and glands, collectively forming the skin microbiota. Together with their genetic material, metabolites, and interactions with the host, these microorganisms constitute the skin microbiome. The term *Stratum microbium* has been proposed to describe this functional microbial layer associated with the skin (Figure 1) [2].

**Figure 2:**
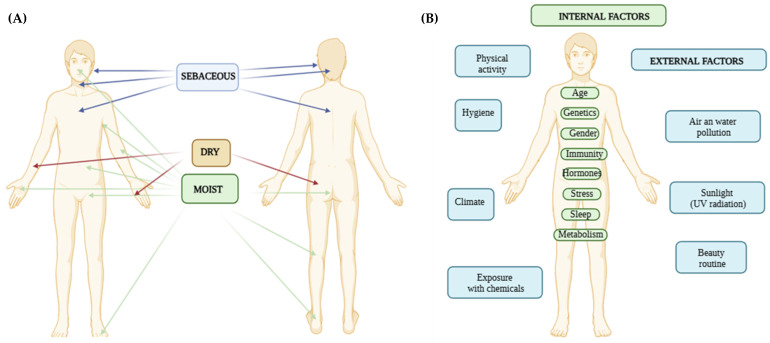
(**A**) Distribution of microenvironments on human skin, (**B**) Internal and external factors influencing the skin microbiome [inspired by [2] and created in BioRender. Łętocha, A. (2026). https://BioRender.com/9lwx9lm (accessed on 3 July 2026)].

Members of the skin microbiota are largely harmless, and in some cases, their presence provides essential functions to the host, making them beneficial for the organism. Symbiotic and commensal microorganisms, which coexist “in harmony” with the host, occupy numerous niches on the skin and protect against colonization by pathogenic organisms as well as harmful substances [2,21,22]. Until recently, the characterization of skin microorganisms relied primarily on culture-based techniques. While many human-associated microorganisms can be cultivated under appropriate laboratory conditions, conventional cultivation methods recover only part of the microbial diversity present on the skin and may favor fast-growing taxa. Consequently, culture-based studies often overrepresent genera such as *Staphylococcus* and *Malassezia* [23,24]. Recent advances in DNA amplification and sequencing technologies have made it possible to bypass cell culture, revolutionizing our understanding of microbial communities inhabiting the skin. DNA sequencing methodologies allow for more precise and accurate microbiome analyses. This approach, based on sequencing the 16S rRNA gene, revealed that most skin-associated bacteria belong to four dominant phyla: *Actinobacteria*, *Firmicutes*, *Proteobacteria* and *Bacteroidetes*. However, taxonomic resolution at the genus and species levels provides a more biologically relevant description of the skin microbiome. The most abundant bacteria include *Cutibacterium* spp. (particularly *Cutibacterium acnes*), *Staphylococcus* spp. (especially *Staphylococcus epidermidis* and *Staphylococcus hominis*), *Corynebacterium* spp., *Micrococcus* spp. and *Acinetobacter* spp., whereas fungi are dominated by *Malassezia* species, mainly *M. restricta* and *M. globosa*. The relative abundance of these microorganisms strongly depends on skin physiology and microenvironmental characteristics [2,21,25,26]. Skin can be divided into three main habitats—moist, sebaceous, and dry—each characterized by a distinct microbial composition (Figure 2A, Table 1) [2,27,28].

In addition to anatomical differences, the variability and richness of the skin’s bacterial flora are influenced by age, sex, season, and ethnic background, as well as stressors such as physical injuries and psychological stress. Environmental factors also contribute to the diversity of the skin microbiome, including climate, temperature, UV radiation, and lifestyle (Figure 2B) [21,29].

## 3. Early Skin Colonization—Factors Influencing Skin Colonization

**Figure 3:**
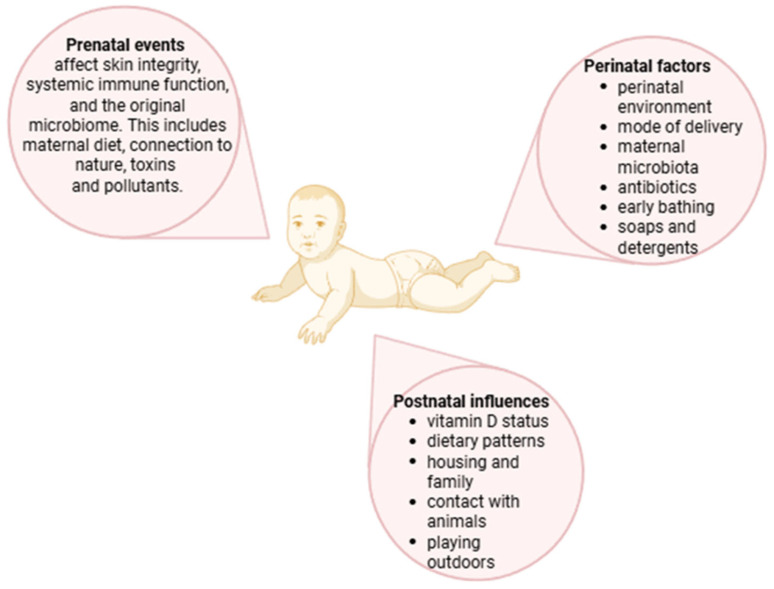
Factors modulating the infant skin microbiome [inspired by [31] and created in BioRender. Łętocha, A. (2026). https://BioRender.com/9lwx9lm (accessed on 3 July 2026)].

The human skin microbiota is established at birth [30]. The newborn skin microbiota begins to develop at birth and is influenced by multiple maternal, neonatal, and environmental factors. Mode of delivery is one of the factors associated with early microbial colonization patterns. Infants born vaginally often show a higher relative abundance of microorganisms commonly found in the maternal vaginal and gastrointestinal microbiota, whereas infants delivered by cesarean section may initially harbour microorganisms associated with maternal skin and the hospital environment. This early colonization process is considered important for the development of immune tolerance to commensal microorganisms. However, these microbial differences can also be influenced by factors such as antibiotic exposure, feeding practices, skin-to-skin contact, hospitalization, and environmental conditions and may change substantially during infancy. Understanding the factors that modulate colonization during this critical period may help prevent potential disease states (Figure 3), as disturbances in skin barrier function and early colonization can predispose to both local and systemic immune dysregulation [31].

The mechanisms of early skin colonization in newborns are complex and depend on interactions between microorganisms, the skin as a barrier organ, and the immune system. Colonization begins during childbirth, when microorganisms are transferred from the mother to the infant. In vaginal delivery, newborns are primarily colonized by bacteria originating from the maternal vaginal and gastrointestinal microbiota, including *Lactobacillus*, *Prevotella*, and *Sneathia* spp. [32,33]. In contrast, infants delivered by cesarean section are mainly colonized by microorganisms from the mother’s skin and the hospital environment, such as *Staphylococcus*, *Corynebacterium,* and *Propionibacterium* [33,34]. Additionally, skin-to-skin contact (SSC) and breastfeeding play a crucial role in the transfer of commensal bacteria and colonization-supporting compounds, such as human milk oligosaccharides [35]. Preterm infants represent a special case, as they exhibit distinct microbiome patterns compared to full-term newborns. In preterm infants, higher abundances of *Staphylococcus*, *Corynebacterium* (phylum *Actinobacteria*), and *Prevotella* (phylum *Bacteroidetes*) are observed. Immune responses to colonizing Staphylococcus species may underlie a common benign condition in newborns—erythema toxicum [36,37]. Commensal bacteria and their products, including staphylococcal lipoteichoic acid and other epitopes, can trigger skin inflammatory responses and enhance immunity. Moreover, protease secretion by commensal *S. epidermidis* prevents colonization by pathogenic *S. aureus* [37].

The newborn’s skin thus represents a dynamic environment for microbial colonization. The factors influencing colonization and their interrelationships are summarized in Table 2.

Newborns possess a set of innate defense mechanisms that participate in the selection and regulation of colonizing microorganisms. Recent discoveries in dermatology highlight the potential importance of antimicrobial peptides (AMPs) in combating microbial skin infections. AMPs additionally act as multifunctional immune effectors, promoting angiogenesis, wound healing, and the production of cytokines and chemokines. In human skin, AMPs such as β-defensins, S100 proteins, and cathelicidins are secreted mainly by keratinocytes, neutrophils, sebocytes, or sweat glands and act selectively against pathogens [41]. These helical peptides bind to lipid membranes and are believed to kill microbes through their ability to disrupt the normal structure of the lipid bilayer [42].

Furthermore, immunological control of microbial colonization on the skin is supported by immunoglobulin A (IgA), present in breast milk [35]. Healthy, breastfed full-term infants receive a mixture of nutrients, bacteria, and antimicrobial proteins—such as carbohydrates, fatty acids, and lactoferrin, along with secretory IgA (sIgA) from breast milk—which shapes the development of their own microbiota [35,43]. Human milk oligosaccharides, glycoproteins, and natural components are also believed to prevent attacks from enteropathogens and stimulate the growth of *Bifidobacterium* [35,44,45]. These compounds can bind directly to the surface of pathogenic microorganisms, while certain milk oligosaccharides prevent pathogens and their toxins from attaching to host cell receptors [46]. In addition, other constituents of human milk—including interleukin-10, epidermal growth factor, transforming growth factor β1, and erythropoietin—may play key roles as regulators of inflammatory responses directed against bacterial pathogens, for example, within the gut [35,44].

Early skin colonization provides an important immunological signal and promotes the development of tolerance to commensals. Commensal skin bacteria, such as *Staphylococcus epidermidis*, induce the differentiation of regulatory T cells (Tregs), which suppress excessive inflammatory responses and protect against the development of inflammatory skin diseases, such as atopic dermatitis [47]. Moreover, *S. epidermidis* contributes to maintaining the skin barrier through the secretion of sphingomyelinase, which is utilized by the host for ceramide synthesis [48,49]. Commensal microorganisms can also enhance barrier functions by stimulating keratinocytes to produce differentiation markers, including filaggrin [50,51], and by increasing AMP expression [50].

Microbial interactions and selective pressures also play a crucial role. Bacteria colonizing the skin participate in biological selection by competing for space and resources. *S. epidermidis* produces bacteriocins—substances acting antagonistically against pathogens such as *Staphylococcus aureus* [47]. Microorganisms also form biofilms, which increase colony stability and favor survival [52]. Life in a shared niche involves not only competition but also cooperation and mutual protection. Some bacteria act synergistically—for example, *Corynebacterium* and *Staphylococcus* have complementary enzymatic abilities, allowing them to survive together on the skin. Studies by Kwaszewska et al. [53] indicate that the lipolytic properties of staphylococci and corynebacteria are clearly complementary. Lipophilic corynebacteria are fatty acid auxotrophs due to their inability to synthesize fatty acids. In most species, these fatty acids are incorporated directly into cellular structures and transformed into corynomycolic acids of the cell wall. Coexistence with active lipolytic staphylococci may be beneficial for them. Moreover, glycerol released after triacylglycerol hydrolysis cannot be utilized by lipophilic corynebacteria, although it serves as a good substrate for staphylococci. According to James et al. [54], this substrate, along with lactic, acetic, or propionic acids, serves as a precursor for volatile fatty acids in non-lipophilic corynebacteria. Additionally, Kwaszewska et al. [53] reported the inability of corynebacteria to utilize macromolecules such as proteins or polysaccharides, whereas staphylococci are highly efficient in this regard, supplying themselves and corynebacteria with products of their enzymatic activity. Although *Corynebacterium* is less tolerant to low pH and salt conditions, cohabitation with *Staphylococcus* provides mutual protection and population stabilization, promoting the development of commensal flora.

Microorganisms colonizing the skin play a key role in the development of immune tolerance and skin barrier functions. Lack of proper colonization is associated with an increased risk of atopy, atopic dermatitis, and other diseases.

## 4. The Influence of the Microbiome on Skin Diseases

**Figure 4:**
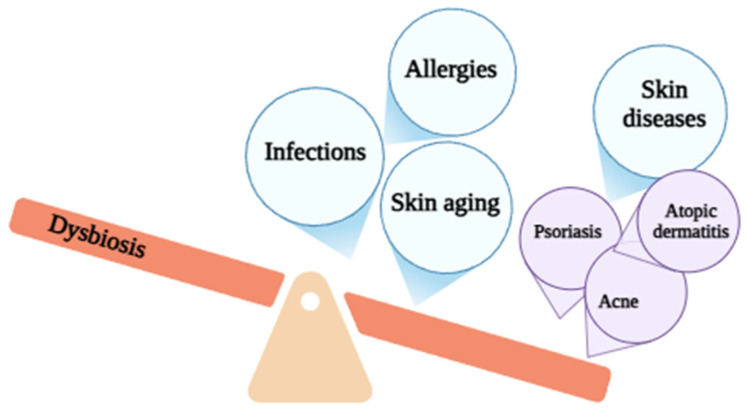
The effects of dysbiosis on human skin [created in BioRender. Łętocha, A. (2026). https://BioRender.com/9lwx9lm (accessed on 03 July 2026)].

The skin is a dynamic organ characterized by continuous renewal processes [55,56]. Alterations in the composition of the stratum corneum can contribute to dysbiosis, affecting both the richness and the relative abundance of commensal microorganisms [31]. Such disturbances in the skin microbiota may, in turn, lead to the development of various dermatological conditions (Figure 4) [29,57].

When the skin barrier function becomes impaired, exacerbation of chronic skin diseases may occur, including atopic dermatitis [16,17,21,27,29,58,59,60], psoriasis [16,17,21,29,59], and acne [16,17,21,29,59,60].

### 4.1. Atopic Dermatitis

Atopic dermatitis (AD) is a long-lasting, relapsing inflammatory condition of the skin, marked by severe itching, dryness, and disruption of the epidermal barrier function [61,62]. The pathogenesis of AD is multifactorial and includes genetic, immunological, and environmental factors, as well as skin microbiome dysregulation. It is noteworthy that the prevalence of AD is significantly higher in industrialized countries and has been systematically increasing for several decades. This suggests that excessive hygiene may disrupt the natural microbial environment of the body, limiting the development of beneficial immune mechanisms and impairing the proper response to pathogen exposure [61,63]. A growing body of research indicates that the skin microbiome plays a key role in both the development and exacerbation of this dermatosis [27,58].

In AD, genetic defects lead to both physical and Th2-dependent immunological impairments of the skin barrier, resulting in increased susceptibility to infections and allergens [27,64]. One of the key epidermal proteins is filaggrin, essential for the production of natural moisturizing factor and maintenance of proper hydration of the stratum corneum. Mutations in the filaggrin gene occur in approximately half of patients with moderate-to-severe AD [65] and are often associated with early-onset disease [66]. Filaggrin deficiency leads to abnormal keratinocyte differentiation and reduced lipid content in the skin [67]. Epidermal lipids, such as ceramides, free fatty acids, and cholesterol, play a critical role in maintaining skin barrier integrity by preventing excessive transepidermal water loss (TEWL) and protecting against the penetration of irritants, allergens, and microbes. Both alterations in composition and reduced levels of these lipids are closely linked to the development of AD [61,68,69].

**Figure 5:**
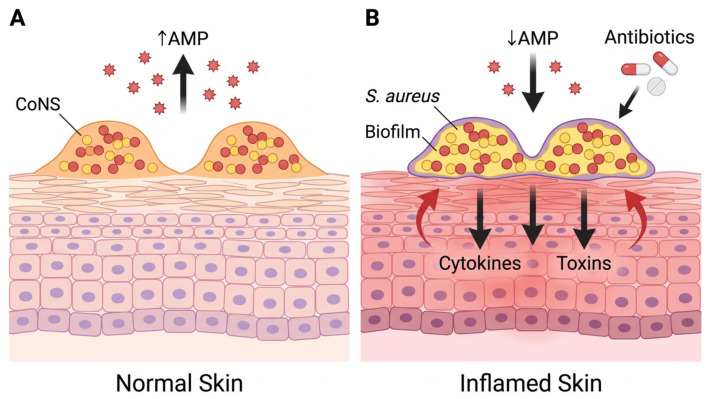
Variability of skin microbiota and biofilm production in the pathogenesis of atopic dermatitis. Arrows indicate increase (↑) and decrease (↓) [inspired by [58] and created in BioRender. Łętocha, A. (2026). https://BioRender.com/anqy4x3 (accessed on 30 June 2026)].

Skin barrier dysfunction is further exacerbated by mechanical damage resulting from chronic scratching of dry and itchy skin [70,71]. Th2 cytokines can inhibit the production of antimicrobial peptides (AMPs) by keratinocytes, such as human beta-defensin-3 and cathelicidins. There may be a link between the sensation of itch in atopic dermatitis and cathelicidins, which can stimulate mast cell degranulation and reduce IL-10 levels in keratinocytes [41,72]. In addition to strengthening the skin barrier, cathelicidins promote cutaneous immunity by increasing the expression and distribution of tight junction components on cell membranes [41,73,74]. Evidence shows that cathelicidin levels are lower in lesional skin of patients with AD and that these levels are correlated with both infection severity in atopic dermatitis and the ability to protect against *Staphylococcus aureus* skin infections. Cathelicidins play an essential role in defense against pathogen colonization, particularly *S. aureus*, thereby contributing to the maintenance of a healthy skin microbiome balance [75]. As the disease progresses, a persistent shift in the skin microbial composition occurs, promoting chronic inflammation and disturbance of homeostasis (Figure 5).

In atopic dermatitis (AD), skin microbiota is notably disrupted, with reduced overall diversity. This imbalance is reflected in diminished levels of genera such as *Cutibacterium*, *Streptococcus*, *Acinetobacter*, *Corynebacterium*, and *Prevotella*, accompanied by a marked expansion of *Staphylococcus*, particularly *S. aureus* [76,77,78,79]. Under normal conditions, in healthy skin, coagulase-negative staphylococci (CoNS) occupy the same ecological niche and limit S. aureus colonization. However, in AD, elevated production of pro-inflammatory cytokines facilitates the proliferation of S. aureus, enabling biofilm formation and allowing it to dominate over other commensal microbes. Consequently, S. aureus further sustains persistent inflammation in eczematous lesions [58].

Reduced microbial diversity is particularly evident during severe disease exacerbations, with reports showing that the *Staphylococcus* composition often decreases to a single *S. aureus* strain [25]. As an opportunistic pathogen, *S. aureus* exhibits a strong ability to adhere to the skin, disrupt epithelial barrier integrity, and activate the host immune system, ultimately driving inflammation [80,81]. While the carriage rate of *S. aureus* in the general population is approximately 20%, significantly higher colonization rates are observed in patients with AD, ranging from 30 to 100% depending on age, disease severity, and sampling and analysis methods [82]. This pathogen produces numerous proteolytic enzymes and toxins and stimulates keratinocytes to increase the expression of endogenous proteases, thereby damaging the skin barrier and facilitating penetration through the stratum corneum [83,84]. Additionally, certain *S. aureus* toxins can trigger type 2 immune responses by activating immune cells and inducing inflammatory mediators such as IL-4, IL-13, IL-22, and TSLP.

It is important to emphasize that in the pathogenesis of AD, not only *Staphylococcus* strains are involved. In a study by Kobayashi et al. [85], Adam17^ΔSox9^ mice developed eczematous inflammation accompanied by a marked alteration in the skin microbiome, with dominance of *S. aureus* and *Corynebacterium* species. Further bacterial inoculation experiments confirmed that inflammation could be enhanced by both *S. aureus* and *C. bovis*. Moreover, *Cutibacterium acnes* (formerly *Propionibacterium acnes*), one of the most common skin commensals and a component of its defense mechanisms [86], may also contribute to disease development. This bacterium increases the cytolytic activity of *S. aureus* by enhancing pro-inflammatory cytokine production [87]. In addition, coproporphyrin III (CIII) produced by *C. acnes* promotes *S. aureus* aggregation and biofilm formation, suggesting synergistic interactions between these two species [88].

On the other hand, there are also reports indicating a lack of direct or even negative correlation between the presence of certain microbes and inflammatory lesion development. Scharschmidt et al. confirmed that early skin colonization in newborns is essential for establishing immune tolerance to commensal microbes [89]. Other studies have shown that coagulase-negative staphylococci *(S. epidermidis*, *Staphylococcus hominis*, and *Staphylococcus lugdunensis*) effectively inhibit *S. aureus* colonization and biofilm formation. In studies by Iwase et al. [90] and Sugimoto et al. [91], serine protease Esp secreted by commensal *S. epidermidis* was shown to inhibit *S. aureus* biofilm formation and colonization. Furthermore, Zipperer et al. [92] reported that nasal isolates of *S. lugdunensis* produce lugdunin, a novel cyclic thiazolidine-containing peptide antibiotic, which prevents *S. aureus* colonization and represents a rare example of a bioactive compound nonribosomally synthesized by human-associated bacteria. A similar effect was demonstrated in a study by Nakatsuji et al. [93], which evaluated both the safety profile and mechanisms of action of *Staphylococcus hominis* A9 (ShA9), a strain derived from healthy human skin, as a candidate for topical treatment of AD. ShA9 was shown to eliminate *S. aureus* from mouse skin and suppress the expression of the *S. aureus* toxin (psmα) responsible for inducing inflammation.

In addition to bacterial commensals, it has also been shown that the protease MgSAP1 secreted by *Malassezia globosa* hydrolyzes *S. aureus* protein A, thereby hindering its biofilm formation [94].

### 4.2. Psoriasis

Psoriasis is a long-term inflammatory disorder with a complex, multifactorial origin, in which impaired immune tolerance to cutaneous microorganisms plays an important role in disease development. It is estimated that approximately 125 million individuals worldwide are affected. The condition substantially influences patients’ quality of life as well as psychosocial well-being. Pathologically, it is characterized by excessive proliferation of epidermal cells alongside inflammation within the dermis. The disease typically begins with an acute inflammatory phase and may subsequently progress into a chronic state [95,96]. Histologically, psoriasis presents with three key features: (1) epidermal thickening resulting from keratinocyte overgrowth, (2) dilation and increased formation of dermal blood vessels, and (3) infiltration and accumulation of inflammatory cells, predominantly within the dermal layer. Clinically, these processes manifest as visible skin lesions, such as scaling plaques, and may also be accompanied by joint or tendon involvement [97].

**Figure 6:**
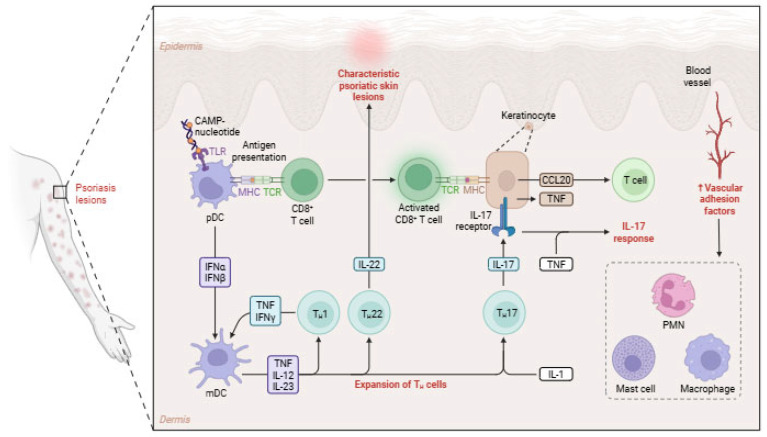
Mechanisms underlying psoriasis and immune system interactions [inspired by [98,101] and created in BioRender. Łętocha, A. (2026). https://BioRender.com/9lwx9lm (accessed on 3 July 2026)].

The pathogenesis of psoriasis involves a complex network of cytokines and their receptors, some of which also represent potential therapeutic targets (Figure 6) [98]. The inflammatory cascade is initiated when plasmacytoid dendritic cells (pDCs) are activated by complexes formed between self-DNA and the antimicrobial peptide cathelicidin (LL-37), released from damaged keratinocytes [98,99]. In parallel, pDCs can present antigens to CD8+ T cells via MHC–TCR interactions, promoting their activation and subsequent migration to the epidermis. In response, pDCs produce IFN-α and IFN-β, which are key mediators in the early phase of the disease. These cytokines subsequently activate myeloid dendritic cells (mDCs) and promote their migration to regional lymph nodes [100]. Once activated, mDCs secrete cytokines such as TNF-α, IL-12, and IL-23, driving the differentiation and expansion of T cells into effector subsets including Th1, Th17, and Th22. IL-1 further contributes to Th17 polarization. Th1 cells produce TNF-α and IFN-γ, whereas Th22 cells secrete IL-22, a cytokine implicated in keratinocyte hyperproliferation and altered epidermal differentiation. These cells enter the circulation and gain the capacity to home to the skin [101,102].

The IL-23/IL-17 signaling pathway plays a central role in psoriatic inflammation. Activated Th17 cells, CD8+ T lymphocytes, and natural killer (NK) cells are major sources of IL-17A, which binds to IL-17 receptors expressed on keratinocytes. This interaction promotes excessive keratinocyte proliferation and contributes to epidermal barrier dysfunction. In the presence of TNF-α, IL-17 further stimulates keratinocytes to produce pro-inflammatory mediators, including TNF-α and the chemokine CCL20, thereby amplifying local inflammation and promoting the recruitment of additional T cells to the skin. IL-22 released by Th22 cells acts synergistically with IL-17 to induce keratinocyte hyperproliferation and abnormal epidermal differentiation, ultimately leading to the formation of characteristic psoriatic plaques. This process is further associated with reduced expression of adhesion molecules and increased production of antimicrobial peptides and chemokines, which facilitate the recruitment of additional immune cells—including Th17 cells, neutrophils, dendritic cells, and macrophages—to the site of inflammation [95].

Psoriasis is the result of interplay between genetic and external factors, wherein the cutaneous microbiota may play a crucial role by responding to external triggers and interacting with the immune system [95,96]. Recent studies have revealed a strong association between skin microbiota and psoriasis. In psoriatic lesions, in contrast to healthy skin, microorganisms are predominantly localized within the epidermal layer. Mechanical damage caused by itch-related scratching may disrupt the skin barrier, allowing certain bacteria—both typical epidermal colonizers and others—to penetrate into deeper dermal layers. In this environment, they can more easily interact with immune cells and trigger both innate and adaptive inflammatory responses. As a consequence, dysbiosis develops. Several studies have reported reduced abundance of taxa such as *Cutibacterium*, *Lactobacillus*, *Burkholderia*, and *Corynebacterium* in psoriatic lesions compared with healthy skin [103]. However, the microbial alterations reported in psoriasis remain heterogeneous across studies. Other investigations have described increased relative abundance of selected bacterial genera, including *Streptococcus*, *Staphylococcus*, and *Corynebacterium*, as well as members of the phyla *Firmicutes* and *Proteobacteria*, in psoriatic skin compared with healthy controls [81,104,105,106,107,108,109,110]. These discrepancies likely reflect differences in study populations, sampling strategies, anatomical sites, disease severity, and analytical methodologies.

Colonization of the skin by *S. aureus* plays an important role in activating the innate immune system, including the induction of antimicrobial peptides and the production of dendritic cell–derived cytokines such as IL-12 and IL-23. This process promotes the expansion of Th17 lymphocytes. These cells subsequently release pro-inflammatory cytokines, including IL-17A, TNF-α, IFN-α, and IL-22, which drive keratinocyte hyperproliferation and enhance immune cell infiltration within the skin, thereby exacerbating psoriatic inflammation [111]. *Streptococcus* species contain components such as M protein and peptidoglycan, which act as superantigens capable of activating T cells. Peptidoglycan can interact with MHC class II molecules and T-cell receptors, leading to a targeted immune response and cytokine secretion. Additionally, T cells in psoriasis patients may cross-react with structurally similar epitopes derived from streptococcal M protein and keratins 6 and 17, which further contributes to disease development [112].

Alterations in the skin microbiome observed in psoriasis are not limited to bacterial populations but also involve fungi. For example, *Malassezia* species can increase the expression of transforming growth factor-β (TGF-β), heat shock protein 70 (HSP70), and integrins in keratinocytes. These changes promote epidermal hyperplasia and cell migration, thereby worsening psoriatic lesions [113]. Furthermore, Malassezia spp. are capable of producing chemoattractants for neutrophils, which may accumulate in large numbers in psoriatic lesions and contribute to the formation of Munro’s microabscesses [114].

*Candida albicans* has likewise been detected in psoriatic skin, particularly in inverse psoriasis. In addition, *Candida*-reactive αβ T cells are known to produce IL-17, which supports the persistence of inflammation and may trigger disease exacerbations [115,116,117,118].

Advances in the study of the skin microbiome in individuals with psoriasis suggest that it may serve as a useful biomarker for predicting therapeutic outcomes and facilitating the selection of individualized treatment strategies. Nevertheless, further research is required to better understand the role of the skin microbiota in the pathogenesis of psoriasis and to evaluate the effectiveness of microbiome-targeted therapeutic approaches.

### 4.3. Acne Vulgaris

**Figure 7:**
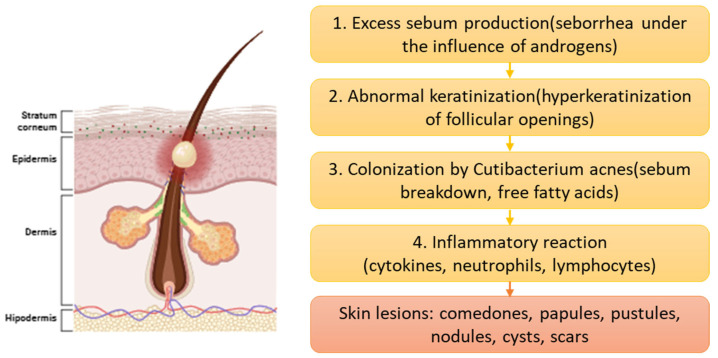
Diagram of acne pathogenesis [inspired by [51] and created in BioRender. Łętocha, A. (2026). https://BioRender.com/9lwx9lm (accessed on 3 July 2026)].

Acne vulgaris is a long-standing inflammatory disorder of the skin with an immunological basis. It is characterized by the presence of both non-inflammatory lesions, such as open and closed comedones, and inflammatory lesions, including papules, pustules, and nodules [119,120]. It affects up to 80% of adolescents, and according to the latest epidemiological data, its prevalence is also increasing in the adult population [119,121,122]. The development of acne is complex, and the interaction between various factors is responsible for the onset and progression of this skin condition, as illustrated in Figure 7.

Excessive activity of the sebaceous glands is considered the primary mechanism in the development of acne; however, growing evidence suggests that the composition of sebum, rather than its overproduction alone, also plays a crucial role [123]. Lipid metabolism disturbances include, among others, a reduction in linoleic acid [124], which exerts a protective effect against comedogenesis, and an increased amount of squalene—a lipid particularly susceptible to oxidation [125]. Oxidized squalene initiates inflammatory cascades by activating lipoxygenase in keratinocytes and stimulating the release of pro-inflammatory cytokines, including interleukin-6 (IL-6) [126]. Excess IL-6 enhances keratinocyte proliferation, leading to hyperkeratinization. Importantly, studies have demonstrated that systemic modulation of lipoxygenase activity reduces the number of inflammatory lesions in patients with acne, further confirming the significance of IL-6 in lipid dysregulation within sebocytes [126].

Disturbances in sphingolipid homeostasis are associated with various skin disorders, including acne vulgaris, where alterations in sphingolipid metabolism contribute to barrier dysfunction and inflammation [123]. Balanced sphingolipid metabolism, particularly the relationship between ceramides and sphingosine-1-phosphate (S1P), is fundamental for maintaining epidermal integrity, regulating immune cell activity, and supporting skin regeneration. A deficiency of sphingolipids leads to excessive follicular keratinization and abnormal desquamation, which contribute to clogged pores and comedone formation in acne patients [127].

Moreover, increased sebum production combined with microcomedone hyperkeratinization creates an anaerobic, lipid-rich environment that favors the proliferation of *Cutibacterium acnes* [128,129]. These bacteria produce biofilm that permeates sebum and acts as a kind of “glue” for corneocytes (mature keratinocytes), facilitating comedone formation [130,131]. Additionally, lipases secreted by *C. acnes* hydrolyze sebum triglycerides into free fatty acids (FFA), which on one hand exacerbate follicular hyperkeratinization and on the other stimulate the production of inflammatory mediators [132,133]. Different FFAs modulate sebocyte function in distinct ways. Particularly important is palmitic acid (PA), which shows strong pro-inflammatory activity. PA interacts with sebocytes by activating TLR2 and TLR4 receptors, leading to increased production of IL-6 and IL-8. Furthermore, PA activates the NLRP3 inflammasome via enhanced reactive oxygen species (ROS) production, resulting in IL-1β release. Elevated PA levels detected in acne lesions indicate its key role in the development of local inflammation [121,134,135,136,137]. Some experimental evidence also suggests that *C. acnes* may stimulate lipid production in sebocytes through the release of soluble factors, contributing to a feed-forward loop between sebum production and bacterial colonization [129,138,139]. In addition, *C. acnes* produces various bacterial molecules and virulence factors—such as enzymes, toxins, and short-chain fatty acids (SCFA)—which amplify pathogenic processes [51,140,141]. Well-characterized virulence factors of this microorganism include lipases, polyunsaturated fatty acid isomerases, glycosidases, sialidases, and CAMP factor proteins [51]. Activation of the cutaneous immune system in response to *C. acnes* has also been demonstrated in vivo [142]. For example, Ashbee et al. reported that serum levels of IgG1 and IgG3 antibodies targeting *C. acnes* were higher in individuals with severe acne compared to those with healthy skin, while IgG2 antibodies specific to *C. acnes* were higher in patients with moderate-to-severe acne compared to those with mild disease [143]. The bacteria also activate pathways related to corticotropin-releasing hormone and Christie-Atkins-Munch-Peterson (CAMP) factors, leading to increased sebum production and an enhanced inflammatory response, including the release of IL-6 and IL-8 in sebaceous glands [120,144,145,146].

In addition to lipid disturbances, bacterial dysbiosis is emerging as a key factor in the pathophysiology of acne vulgaris, with changes in the skin microbiome contributing to the inflammatory phenotype of the disease [51]. In recent years, increasing attention has been paid to strain-level differences in *Cutibacterium acnes* and the interaction between the skin microbiota and the immune system. Despite evidence that *C. acnes* contributes to acne development, no consistent difference in the relative abundance of this bacterium has been detected between acne patients and healthy individuals [147,148,149]. In acne pathogenesis, the critical factor is not a quantitative increase in bacterial load but rather qualitative changes in microbiome composition, including the predominance of certain *C. acnes* phylotypes (particularly IA1 and IA2) and an imbalance of other commensals. *C. acnes* is classified into several phylogenetic subtypes: IA1, IA2, IB, IC, II, and III [150]. In a genetic analysis conducted by Shimokawa et al. [151], the distribution of phylotypes was compared between acne patients and healthy controls. The most frequently detected subtype was IA1, consistent with earlier findings [152]. Subtype IA1, isolated from acne lesions, shows high pathogenic potential [86,153,154]. In contrast, type III displays distinct characteristics: its prevalence differed significantly between groups, being present on healthy skin but absent from typical acne sites. Type III lacks toxic factors, and its morphology and metabolic pathways differ from those of types I and II [155].

The coexistence of *C. acnes* with other microorganisms may also be relevant to acne. *C. acnes* possesses various strategies for niche competition within hair follicles, including the production of bacteriocins (acnecin and cutimycin) and propionic acid [141]. Besides the extensively studied *C. acnes*, other microorganisms, such as the most abundant commensal skin fungi, Malassezia spp. and *Staphylococcus* species, are associated with acne [77,156,157]. Dagnelie et al. analyzed the impact of dysbiosis between *Cutibacterium acnes* and Staphylococcus epidermidis. Human skin explants were cultured with varying ratios of the two bacteria—predominance of *C. acnes*, predominance of *S. epidermidis*, or balance (1:1). Dysbiosis induced a stronger inflammatory response compared with balance. Notably, *S. epidermidis* strongly induced IL-6 secretion, a key mediator of innate pro-inflammatory immune responses [158]. However, Xia et al. [159] found that lipoteichoic acid (LTA) generated by *S. epidermidis* can inhibit *C. acnes* proliferation and decrease TLR2 protein expression in keratinocytes [142]. Therefore, further studies are needed to clarify the role of S. epidermidis in acne development and resolve these discrepancies. In contrast, O’Neill et al. [160] demonstrated that the *Staphylococcus capitis* strain (*S. capitis* E12) effectively and selectively inhibited *C. acnes* growth, acting more strongly than commonly used antibiotics. This mechanism was based on modulins secreted by *S. capitis* E12, which acted selectively against *C. acnes* without disturbing the rest of the skin microbiota. Understanding the role of *Staphylococcus* species in acne pathogenesis opens new possibilities for therapeutic interventions [157].

Similarly to *Cutibacterium acnes*, the lipophilic properties of *Malassezia* spp. suggest its potential involvement in acne pathogenesis. This association is supported by favorable responses to antifungal treatment in patients with acne resistant to antibiotic therapy. Hu et al. [161] demonstrated that antifungal therapy in patients with resistant papules and pustules resulted in a significant reduction in acne lesions, and in some cases, complete resolution after discontinuation of antibiotics. Moreover, *Malassezia* shows increased abundance in young individuals with acne [162], elevated lipase activity, and the ability to stimulate immune responses in PBMCs (peripheral blood mononuclear cells) and keratinocytes [163,164]. Despite these observations, clear evidence directly confirming a causal relationship between *Malassezia* and acne development is still lacking.

### 4.4. Adjunctive Therapies for Microbiome-Associated Skin Dermatoses

**Figure 8:**
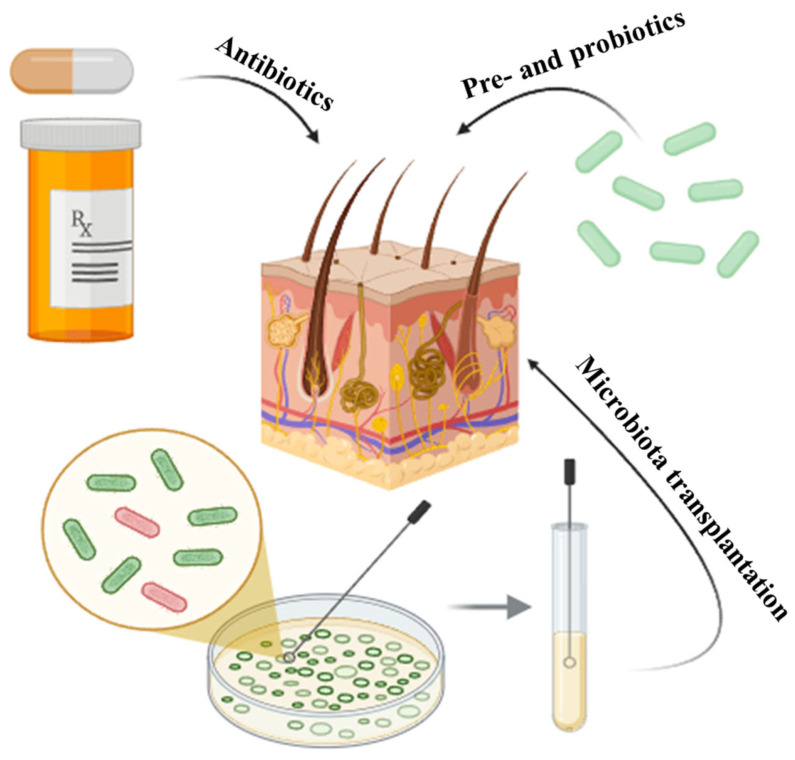
The major concepts to regenerate the human skin microbiota [created in BioRender. Łętocha, A. (2026). https://BioRender.com/9lwx9lm (accessed on 3 July 2026)].

When skin barrier integrity is impaired and skin microbiome homeostasis is disturbed, several approaches, including antibiotic therapy, prebiotic and probiotic supplementation, and microbiota transplantation, have been investigated as potential strategies to support restoration of microbial balance (Figure 8).

One possible approach to combating skin microbiome-related diseases is microbiome transplantation. Myles et al. [165], for the first time ever, performed a local microbiome transplant using *Roseomonas mucosa* isolates from healthy donors for the treatment of atopic dermatitis. As a result, they observed improved clinical scores of atopic dermatitis (less itching, improved skin) and reduced *S. aureus* colonization in some patients. However, the study also had limitations, such as the small number of patients (10 adults and 5 children were included in the open-label study). In a randomized phase 1 clinical trial, *Staphylococcus hominis* A9 (Sh A9), a bacterium isolated from healthy human skin, was used as a topical therapy for atopic dermatitis. *S. hominis* was applied to the forearms of 54 patients with atopic dermatitis for 8 days and resulted in a significant reduction in *S. aureus* compared to vehicle [93]. Given the constant interplay between the gut and skin microbiome, the frequent association of psoriasis with inflammatory bowel disease, and the impact of gut dysbiosis on the course of psoriasis, the use of fecal microbiota transplantation (FMT) in patients with psoriasis has been considered. Yin et al. successfully performed FMT in a patient suffering from psoriasis and irritable bowel syndrome [166]. Despite promising results, there are still many challenges and limitations (risk of transmission of undesirable microorganisms, individual microbiome variability) that hinder the widespread use of skin microbiota transplantation in the treatment of dermatological diseases. Therefore, long-term clinical trials on safety and efficacy are needed before this method becomes a therapeutic option [167].

Another approach currently used is antibiotic therapy. Topical antibiotics, such as clindamycin and erythromycin, are often used to combat *C. acnes* and other acne-related bacteria. They inhibit bacterial protein synthesis, reducing the bacteria that contribute to acne, but their broad-spectrum action can disrupt the skin microbiome, affecting both harmful and beneficial bacteria [157]. In this case, the problem of overuse of antibiotics also arises. While antibiotics were initially, and remain, an important milestone in the treatment of bacterial infections, their widespread use has become a major public health concern due to the emergence of numerous antibiotic-resistant strains of microorganisms, making the treatment of infections increasingly difficult and, in some cases, nearly impossible. A study of Korean acne patients revealed high rates of resistance of *S. epidermidis* to tetracycline (31%), doxycycline (27%), clindamycin (33%), and erythromycin (58%) [168]. Another study conducted in Jordan showed that 35% of *S. aureus* isolates and 25% of *S. epidermidis* isolates were resistant to antibiotics [169]. Another study conducted in France involving 1472 hospitalized patients showed that all *Cutibacterium* strains remained susceptible to amoxicillin, ceftriaxone, vancomycin and moxifloxacin [170]. However, it was noted that 15% of *C. acnes* strains were resistant to erythromycin, 4.1% to clindamycin, and 2.2% to tetracycline. These results indicate the high adaptability of this microorganism, particularly in the context of forming biofilms on the skin surface and within the sebaceous glands [171]. In biofilm structures, *C. acnes* gains protection against antibiotics because its matrix limits the penetration of drugs into the colony [172]. Additionally, bacteria within the biofilm communicate through quorum sensing and exhibit reduced metabolic activity, making effective antibiotic treatment even more difficult [173]. These findings underscore the growing concern about antibiotic resistance in the treatment of skin diseases, emphasizing the need for alternative therapeutic approaches and more judicious use of antibiotics to maintain their effectiveness. In this context, when skin dysbiosis and microbiome diversity are disrupted, prebiotics and probiotics are a promising avenue, as they can act as modulators to restore microbial balance [174].

## 5. Pre- and Probiotics in Cosmetics

Prebiotics and probiotics are widely used in the food, dietary supplement, and pharmaceutical industries due to their attributed health benefits. In the case of food and pharmaceutical products, probiotics are applied to regulate the gut microbiome and to combat antibiotic resistance. The rise in antibiotic resistance and associated side effects has prompted the search for alternative therapies. Antibiotic resistance poses a serious threat to modern medicine and global public health. It is estimated that antibiotic resistance accounts for approximately 1.3 million deaths worldwide [175,176], and projections suggest that this number will increase to 10 million deaths globally by 2050 [177]. Recent research indicates that probiotic supplementation shows considerable therapeutic potential, as it can help reestablish gastrointestinal microbiota homeostasis and reduce disruptions caused by antibiotic use while also indirectly inhibiting the growth of *Helicobacter pylori* (*H. pylori*) [178], a bacterium responsible for gastritis and peptic ulcer disease of the stomach and duodenum. Initiated by Grice, E. A. and Segre, J. A. Reference [2] and currently continued by research groups worldwide, studies indicate that probiotics may not only serve as an alternative in the fight against antibiotic resistance or peptic ulcer disease but may also be beneficial in the treatment of skin disorders.

The growing interest in microorganisms colonizing the human body—not only those causing infections—has led to numerous studies aiming to regulate microbiome composition and to investigate how its modulation impacts human health. The global market for pre- and probiotics continues to expand, partly attributed to consumer awareness of their health benefits [19,60,179]. This also includes cosmetic applications, where the probiotic market is expected to grow by 12% over the next decade, driven largely by North America [179]. Current definitions of these terms have been developed primarily based on their use as dietary supplements [180].

### 5.1. Prebiotics

The definition of prebiotics has evolved considerably over the last two decades. According to the current consensus statement of the International Scientific Association for Probiotics and Prebiotics (ISAPP), a prebiotic is defined as “a substrate that is selectively utilized by host microorganisms conferring a health benefit” [181]. This updated definition broadened the original concept of prebiotics beyond non-digestible dietary carbohydrates acting exclusively in the gastrointestinal tract. It acknowledges that prebiotics may include different classes of compounds, may act at body sites other than the gut, and can be incorporated into various products, including topical formulations [181].

In the context of skin health, prebiotics are used to support beneficial members of the skin microbiota and to promote a balanced microbial ecosystem. By selectively stimulating commensal microorganisms, prebiotics may contribute to maintaining skin homeostasis, strengthening barrier function, and reducing the risk of dysbiosis-associated skin disorders [19,182]. Interest in topical prebiotics has increased together with growing recognition of the role of the skin microbiome in conditions such as atopic dermatitis, acne vulgaris, and psoriasis.

At present, fructooligosaccharides (FOS) and galactooligosaccharides (GOS) are the most commonly recognized prebiotics, as demonstrated in numerous studies evaluating their biological activity [181]. However, plant-derived polyphenols have recently been proposed as another group of compounds with potential prebiotic properties. It is estimated that the majority of dietary polyphenols (approximately 90–95%) are not absorbed in the small intestine and instead reach the colon, where they are extensively metabolized by the gut microbiota [183]. Growing evidence indicates that the beneficial health effects associated with polyphenol intake are largely mediated by microbial transformation and the resulting metabolites, rather than by the original compounds themselves [184]. These observations broaden the definition of prebiotics beyond traditional non-digestible carbohydrates such as FOS and GOS. Nevertheless, further studies are required to confirm their effectiveness and formally establish their status as prebiotic substances.

Despite increasing interest in microbiome-targeted skincare, the mechanisms through which prebiotics influence the skin microbiota remain less well characterized than those of probiotics. Nevertheless, prebiotics represent a promising strategy for supporting microbial balance and maintaining healthy skin function.

### 5.2. Probiotics

According to the widely accepted FAO/WHO definition, “probiotics are live microorganisms which, when administered in adequate amounts, confer a health benefit on the host” [12,28]. The most common probiotics are representatives of the genera *Lactobacillus* and *Bifidobacterium*. In contrast, components derived from *Lactococcus*, *Streptococcus*, *Leuconostoc*, *Pediococcus*, and *Saccharomyces* are used much less frequently [185]. It should be noted that probiotic activity is strain-specific rather than species-specific [28,186]. Moreover, different strains may exhibit distinct probiotic properties, and the parameters of one strain cannot be extrapolated to another, even if they belong to the same species [28,185]. This fact is of particular importance when selecting the appropriate strain for specific applications.

In the field of cosmetics, however, the term “probiotic” is frequently used more broadly than its scientific definition allows. Because maintaining viability, stability, and microbiological safety of live microorganisms in cosmetic products is technically challenging and often incompatible with conventional preservation systems, most cosmetic formulations do not contain live probiotic microorganisms [13,60]. Consequently, many commercially available products marketed as “probiotic cosmetics” actually contain non-viable microorganisms, lysates, fermentation products, or microbial metabolites rather than true probiotics as defined by ISAPP [60,180,187].

Therefore, it is important to distinguish between true probiotics (live microorganisms) and probiotic-derived cosmetic ingredients, which are often referred to in the cosmetics literature as “probiotic ingredients” despite not meeting the formal definition of probiotics [180,187].

Among cosmetic ingredients derived from probiotic microorganisms, four categories can be distinguished [13,185,186]:

- Fermentation products—probiotic bacteria are grown in a specific culture medium, later removed, but the solution contains their metabolites such as amino acids, vitamins, and antioxidants.
- Cell lysates—bacteria are not filtered out of the culture medium but are inactivated; the solution thus contains structural components of their cells.
- Tyndallized (heat-killed) microorganisms—bacteria are killed by heating before being introduced into the formulation. Although no longer viable, their cellular components and metabolites exert immunomodulatory effects and inhibit pathogen development.
- Live probiotic microorganisms—the only category that fully complies with the scientific definition of probiotics.

Among these categories, fermentation products are currently the most common ingredients used in cosmetic formulations [60]. However, strictly speaking, fermentation products, cell lysates, and tyndallized microorganisms should not be classified as probiotics because they do not contain viable microorganisms. According to the current ISAPP consensus, such preparations are more closely related to the concept of postbiotics.

In 2021, the International Scientific Association for Probiotics and Prebiotics (ISAPP) defined postbiotics as “a preparation of inanimate microorganisms and/or their components that confers a health benefit on the host” [187]. According to this definition, postbiotics are complex preparations containing non-viable microorganisms and their structural components. These preparations may include a wide range of bioactive substances, such as enzymes, peptides, teichoic acids, surface-associated proteins, polysaccharides, and organic acids, which can exert beneficial effects on host health [188,189,190,191].

Importantly, purified microbial metabolites alone are not considered postbiotics under the ISAPP definition, although they may contribute to the biological activity of postbiotic preparations [187]. Therefore, microbial lysates, heat-killed microorganisms, and preparations containing inactivated microbial cells can be classified as postbiotics, whereas isolated fermentation products or individual microbial metabolites should be referred to as microbial-derived compounds or microbial metabolites.

Comparing these two concepts, the distinction becomes clear: probiotics are “live microorganisms,” whereas postbiotics are “inanimate microorganisms.” However, despite its use in the food industry, this term is rarely applied in the context of cosmetic raw materials, where metabolites such as lysates, fermentation products, or tyndallized bacteria are still referred to as “probiotics” or “probiotic raw materials” [60,192]. The literature uses a range of terms for such substances: probiotic raw materials, non-viable probiotics, heat-killed probiotics, or tyndallized probiotics [180]. Research indicates that even such forms containing inanimate microorganisms may exert beneficial biological effects [193]. When used as cosmetic ingredients, they have been shown to improve skin condition by moisturizing, alleviating inflammation and UV-induced photodamage, and improving atopic dermatitis–affected skin [19,31,56,182,193,194,195,196,197,198]. Thus, both live probiotics and probiotic-derived ingredients (including lysates, fermentation products, and other postbiotic preparations)—may help prevent inflammatory and allergic skin conditions [199]. Clinical studies demonstrated that the application of *Bifidobacterium longum* lysate is effective in treating sensitive and dry skin [194,195]. In ex vivo studies with *B. longum* extract, Guéniche et al. [194] reported statistically significant improvements compared with placebo in inflammation-related parameters such as reduced vasodilation, edema, and TNF-α release. Furthermore, volunteers applying creams containing bacterial extracts showed significant decreases in skin sensitivity and dryness after 29 days of treatment. In an animal model of atopic dermatitis, Kim et al. [193] demonstrated that two weeks of treatment with *Lactobacillus sakei* probio 65 (both live and heat-killed forms) significantly inhibited the development of AD-like skin lesions, as evidenced by decreased serum immunoglobulin E levels compared with the control group. Studies by Jung et al. [196] on reconstructed epidermis (Keraskin™) confirmed that *Lactobacillus rhamnosus* lysates positively affect skin barrier function by increasing the expression of tight junction proteins (claudin, occludin) and barrier proteins such as filaggrin. Pretreatment with lysates also reduced the cytotoxicity of sodium lauryl sulfate (SLS) and mitigated desmosome damage induced by SLS. In a study by Khmaladze et al. [197], the probiotic strain *L. reuteri* DSM 17938 was tested in topical applications using two models: ex vivo (anti-inflammatory and barrier-supporting effects) and in vitro (antimicrobial activity). Both live and lysed forms reduced IL-6 and IL-8 pro-inflammatory activities. Moreover, the live form demonstrated antibacterial effects against skin pathogens (*S. aureus*, *S. pyogenes* M1, *C. acnes* AS12, and *P. aeruginosa*), whereas the lysate did not. These results suggest that live *L. reuteri* DSM 17938 may be useful in managing photoaging, bacterial overgrowth, and skin hydration, while lysates may be more suitable in contexts requiring anti-inflammatory activity. Therefore, the use of live microorganisms appears to be highly promising in the context of topically applied skin formulations [28].

Despite the fact that cosmetic products increasingly claim to contain “probiotics”, which are usually fermentation-derived products, there is great interest in the use of live bacteria for managing dry and sensitive skin or inflammatory skin diseases [197,200]. For example, in a clinical study, Peral et al. [201] demonstrated that topical application of live *Lactobacillus plantarum* reduced inflammation and accelerated wound healing in patients with chronic venous leg ulcers. In 14 diabetic and 20 non-diabetic patients, topical application of *L. plantarum* to lesions (25–60 cm^2^) resulted in wound cleansing, granulation tissue formation, and complete healing within 30 days in 43% of diabetic patients and 50% of non-diabetic patients. Biopsies collected after 10 days showed decreased proportions of polymorphonuclear, apoptotic, and necrotic cells, as well as modulation of IL-8 production (initially increased after 5 days, then decreased after 10 days), ultimately accelerating wound repair. In a clinical trial by Butler et al. [7], an ointment containing the *L. reuteri* strain DSM 17938 was shown to significantly improve the SCORAD score in adult patients with atopic dermatitis after 4 and 8 weeks of continuous use. Recent studies have also shown that topical probiotics can alleviate acne by increasing ceramide production, reducing inflammation, and protecting against pathogens [202]. A comparison of *L. paracasei* lotion with 2.5% benzoyl peroxide demonstrated that both effectively reduced inflammation and erythema, but adverse events were less common with the probiotic lotion [203]. Although the evidence comes from limited studies, the results are promising. Importantly, the observed benefits of microbiome modulation suggest that similar mechanisms may also play a key role in protecting skin from premature aging and UV-induced damage. Although the evidence comes from limited studies, the results are promising.

Importantly, the observed benefits of microbiome modulation indicate that similar mechanisms may also play a key role in protecting skin from premature aging and UV-induced damage.

## 6. The Role of Biotics in the Context of Anti-Aging

Skin aging can be divided into two main forms: chronological aging and photoaging [204,205]. Chronological aging is mainly driven by intrinsic factors, whereas photoaging results from external factors, primarily UV radiation [206]. Although the causes of these two types of skin aging differ, their molecular mechanisms show significant similarities. In the context of anti-aging interventions, strengthening the epidermal barrier, stimulating regeneration and collagen synthesis, and regulating skin pH are particularly important.

Growing evidence suggests that microbiome-targeted interventions may contribute to healthy skin aging through improved barrier function, enhanced hydration, reduced oxidative stress, and modulation of inflammatory pathways. However, it is important to distinguish between the effects of live probiotics and those of postbiotics or probiotic-derived ingredients, as many studies conducted in dermatology and cosmetic science have investigated non-viable microbial preparations rather than live microorganisms.

### 6.1. Postbiotics and Probiotic-Derived Ingredients in Anti-Aging Applications

Most microbiome-related anti-aging studies performed in dermatology and cosmetic science have investigated postbiotics and probiotic-derived ingredients rather than live probiotic microorganisms. These preparations include bacterial lysates, fermented extracts, heat-killed (tyndallized) microorganisms, and microbial metabolites [188].

Postbiotics and probiotic-derived ingredients support the production of ceramides and structural proteins (e.g., filaggrin), which improve epidermal barrier integrity and reduce transepidermal water loss (TEWL) [207]. Studies conducted by various research groups have demonstrated the significant anti-aging effects of postbiotics. For example, Ogawa et al. [208] in a randomized, placebo-controlled clinical trial, examined the role of heat-killed *Lactobacillus brevis* SBC8803 (SBL88™) in improving skin hydration and showed that oral administration of SBL88™ reduced TEWL, proving its potential to enhance skin hydration and reduce dryness [207,209]. Jung et al. [196] studied *Lactobacillus rhamnosus* (LR) lysates in a reconstructed human epidermal model (Keraskin™). Application of LR lysate increased the expression of tight junction proteins (claudin-1 and occludin) as well as skin barrier proteins (loricrin and filaggrin), both at the protein and mRNA levels. Moreover, LR lysate demonstrated beneficial effects on skin barrier function by reducing SLS-induced cytotoxicity and skin permeability in Keraskin™, and it also protected desmosomes from SLS-induced damage. In an in vitro study, Im et al. [210] measured hyaluronic acid (HA) content via ELISA and found that HA, a key molecule in skin hydration [211], increased after treatment with tyndallized *Lactobacillus acidophilus* IDCC 3302. Wang et al. [212] (in vitro study) reported that topical application of fermented *Bifidobacterium* lysate enhanced the expression of genes such as FLG (filaggrin), LOR (loricrin), IVL, TGM1, AQP3, and antimicrobial peptides (CAMP, hBD-2) in HaCaT keratinocytes, suggesting improved barrier integrity and immunomodulatory mechanisms. In yet another study, Kim et al. [213] observed increased sphingomyelinase activity and, consequently, higher ceramide production after treatment with *L. rhamnosus* IDCC 3201 lysates.

Another mechanism by which microbiome-derived preparations act on the skin involves stimulating fibroblasts to produce type I collagen. In an in vitro study by Huuskonen et al. [214], *Bifidobacterium animalis ssp. lactis* strains Bl-04 and B420 most effectively supported type I procollagen production and maintained a favorable MMP-1 to type I collagen ratio under pro-inflammatory (TNF-α) stimulation. Importantly, it was not the cell lysates but rather metabolites produced by these bifidobacteria that significantly reduced the levels of several pro-inflammatory cytokines (IL-6, IL-8, and TNF-α) during inflammatory provocation. These findings suggest that metabolites produced by Bl-04 and B420 may positively affect collagen homeostasis in the skin and mitigate inflammatory responses.

Collectively, these findings indicate that postbiotics and probiotic-derived ingredients may support skin hydration, barrier integrity, and collagen homeostasis, thereby contributing to anti-aging strategies.

### 6.2. Probiotics in Anti-Aging Applications

Probiotic microorganisms employ various mechanisms, such as lowering pH, to maintain skin health and inhibit the growth of pathogens. The acidic environment of the skin (<5.0) discourages bacterial colonization and provides a moisture barrier through the absorption of water by amino acids, salts, and other substances in the acid mantle [215]. In an in vitro study by Lambers et al. [216] demonstrated that the commensal skin species *Staphylococcus epidermidis* showed better growth at pH 4.7 in the presence of lactate buffer compared to neutral pH (pH 7). In contrast, the growth of the pathogenic *Staphylococcus aureus* was strongly inhibited under these conditions [216]. This suggests that in the natural, slightly acidic environment of the skin, *S. epidermidis* may gain an advantage over *S. aureus* due to its ability to grow in the presence of lactic acid. Furthermore, this study also showed that individuals with skin pH < 5.0 exhibited significantly less scaling and higher hydration levels compared to those with skin pH > 5.0. Another mechanism of probiotics is fermentative metabolism, which involves the production of acidic molecules (e.g., lactic acid), thereby acidifying the surrounding environment [217].

In human dermal fibroblasts and a hairless mouse model, Ra et al. [218] evaluated the effect of live *Lactobacillus plantarum* HY7714 on skin hydration in human dermal fibroblasts and hairless mice. In Hs68 cells, *L. plantarum* HY7714 not only increased mRNA levels of serine palmitoyltransferase (SPT) but also reduced ceramidase mRNA levels, leading to improved ceramide levels. Ceramides play a crucial role in maintaining skin barrier integrity and hydration [219,220,221]. Better skin hydration translates into smoother wrinkles and improved elasticity. However, studies on the effects of live probiotic bacteria and cosmetic formulations on skin condition remain limited.

Meanwhile, UV radiation plays a key role in photoaging, activating numerous signaling pathways that affect, among others, collagen synthesis and the production of matrix metalloproteinases (MMPs) [222]. In this context, the influence of bacterial forms, particularly probiotics, on the activity of kinases involved in these processes has gained increasing attention, as it may beneficially affect both types of skin aging.

Additionally, UV radiation accelerates telomere shortening, supporting the concept that chronological aging and photoaging often overlap. This results in the accumulation of DNA damage in skin cells, which is repaired through two main mechanisms: nucleotide excision repair (NER) and base excision repair (BER) [223,224]. DNA damage induced by reactive oxygen species (ROS) is mainly repaired via BER, whereas direct DNA damage caused by UV radiation is primarily repaired by NER [225].

## 7. The Role of Biotics Against Ultraviolet (UV) Radiation

**Figure 9:**
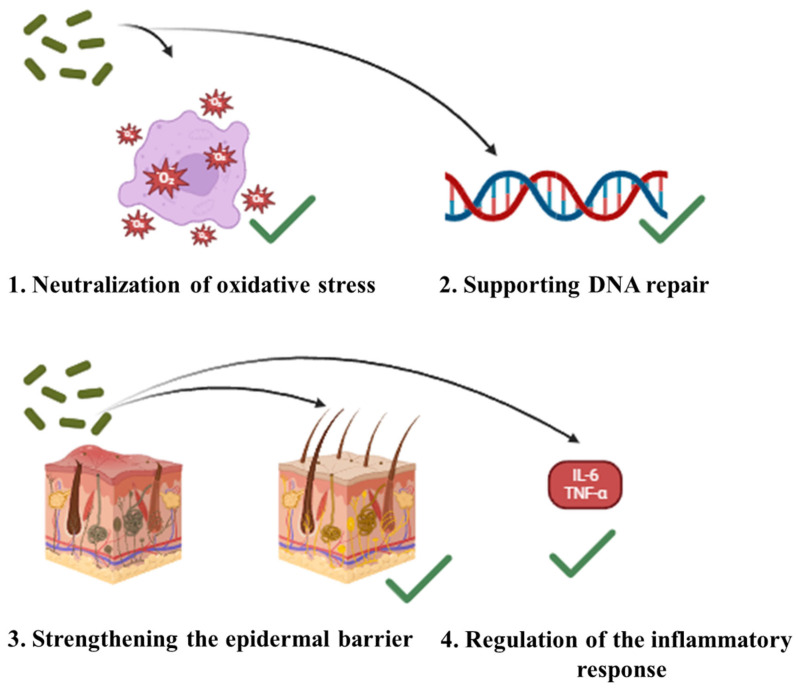
Defense mechanisms of probiotics against UV [created in BioRender. Łętocha, A. (2026). https://BioRender.com/9lwx9lm (accessed on 3 July 2026)].

Ultraviolet (UV) radiation, particularly the UVB (290–315 nm) and UVA (315–400 nm) ranges, is one of the most significant environmental stressors for human skin. Exposure to UV leads to DNA damage, oxidative stress, disruption of the epidermal barrier function, as well as induction of inflammation and photoaging [182]. In recent years, there has been growing interest in the use of skin probiotics as potential agents to help protect the skin against the harmful effects of UV (Figure 9).

### 7.1. Postbiotics and Probiotic-Derived Preparations Against UV-Induced Skin Damage

Ultraviolet (UV) radiation induces the generation of reactive oxygen species (ROS), leading to lipid peroxidation and damage to cellular structures. Numerous in vitro studies have demonstrated that postbiotic preparations possess antioxidant properties, providing a certain degree of protection against oxidative stress [226,227,228]. Experimental evidence of the antioxidant activity of heat-killed *Lactobacillus acidophilus* KCCM12625P (AL) was provided by Lim et al. [60,229]. It was found that in human keratinocytes and human dermal fibroblasts (HDF) exposed to UV light, AL regulated ROS and MMP (matrix metalloproteinase) levels. AL was also able to reduce UV-induced melanin production in B16F10 mouse melanoma cells. Furthermore, studies by Im et al. [230] showed that heat-inactivated *Lactobacillus acidophilus* could reverse UV-induced skin damage by lowering MMP expression and enhancing skin antioxidant mechanisms.

Prolonged exposure to UV radiation can induce not only oxidative stress but also DNA damage, inflammation, and apoptosis [231,232]. Xu et al. [233] demonstrated that heat-killed *Lacticaseibacillus paracasei* (PL) significantly reduced DNA damage in NHDF cells and B16F10 melanocytes after UVB exposure, which was associated with a decrease in cyclobutane pyrimidine dimer (CPD) levels and an increase in the level of XPA protein, a key component of the NER pathway. These findings indicate the potential role of postbiotic preparations in stimulating DNA repair pathways.

Postbiotic preparations may also modulate inflammatory responses induced by UV radiation. Kimoto-Nira et al. [234] reported that an extract obtained from heat-killed *Lactococcus lactis* H61 reduced UV-induced interleukin-8 (IL-8) levels in pretreated cells while also absorbing electromagnetic radiation in the UVB range.

Other studies suggest that postbiotics may strengthen the epidermal barrier and support skin regeneration following UV exposure. Mondadori et al. [235] investigated the potential of heat-treated probiotics (Skinbac™) as protective effects against ultraviolet radiation, specifically both UVA- and UVB-related injury, using the human keratinocyte cell line HaCaT. As expected, exposure to UVA and UVB reduced cell viability. However, 24 h treatment of cells with heat-treated Skinbac™ probiotics supported regeneration and significantly increased survival. This effect was further confirmed in in vivo studies using a three-dimensional human skin model (Phenion^®^ Full-Thickness Skin Model). Similarly, Shaheen et al. [236] suggested significant radioprotective effects of hyaluronic acid (HA) produced by *Enterococcus durans* K11 and *Lactiplantibacillus plantarum* St3 against UVB radiation in human keratinocytes. In their study, HA obtained from strains K11 and St3 significantly improved the viability of UVB-exposed keratinocytes to 91.3% and 91.4%, respectively, compared to the control group (76%). These findings suggest significant radioprotection of human keratinocytes against UVB radiation [236]. Another study by Fu et al. [237] showed that *Rhodiola rosea* fermented with *Lactobacillus plantarum* significantly reduced cellular matrix metalloproteinase activity, thereby increasing collagen and elastin content and alleviating UVA-induced photoaging.

### 7.2. Probiotics Against UV-Induced Skin Damage

Studies evaluating the photoprotective effects of live probiotic microorganisms are comparatively less numerous; however, available evidence suggests that viable probiotic strains may help protect the skin through antioxidant, anti-inflammatory, and immunomodulatory mechanisms.

In an in vitro study by Wang et al. [238] pretreatment with *L. plantarum* ZLP001 reduced ROS production and malondialdehyde concentration in cells while increasing mitochondrial membrane potential compared to H_2_O_2_ treatment alone, suggesting that *L. plantarum* ZLP001 promotes the maintenance of cellular redox homeostasis. Moreover, *L. plantarum* ZLP001 regulated the expression and production of certain antioxidant enzymes, thereby activating the antioxidant defense system.

Both UVA and UVB radiation can cause photochemical damage by inducing ROS production and oxidative lesions [239], and ROS can directly or indirectly lead to DNA damage. DNA lesions caused by UV radiation primarily affect the electronic structure of DNA, promoting chemical reactions between bases, especially thymine dimerization (cyclobutane derivatives) [240]. Direct absorption of UV radiation by DNA can result in adducts between adjacent pyrimidine nucleotides, forming cyclobutane pyrimidine dimers (CPD) and pyrimidine (6–4)pyrimidone photoproducts [(6–4)PP] [241]. Probiotics may indirectly enhance the skin’s ability to repair UVB-induced DNA damage through the activation of nucleotide excision repair (NER) pathways and modulation of the expression of DNA damage response genes such as XPC and TP53. Although there is currently no conclusive evidence for the direct upregulation of XPC, p53, or other NER components by live probiotics, similar effects have been observed in studies involving bioactive compounds such as silibinin [242] or green tea polyphenols [243]. These substances have been shown to activate the p53-dependent DNA repair pathway, leading to increased expression of XPC and XPA genes and accelerated elimination of cyclobutane pyrimidine dimers (CPD).

Another mechanism of probiotic action is the modulation of the inflammatory and immune responses. Probiotics have the ability to suppress excessive inflammatory reactions after UV exposure, for example, by reducing levels of pro-inflammatory cytokines (IL-1β, IL-6, TNF-α) and inducing anti-inflammatory cytokines (IL-10). In a randomized, placebo-controlled clinical trial, Peguet-Navarro et al. [244] showed that oral supplementation with *Lactobacillus johnsonii* protected against UV-induced suppression of contact hypersensitivity, reduction in epidermal Langerhans cell density, and increased IL-10 levels in serum. In the absence of UV exposure, probiotic bacteria exerted no detectable effect on the skin immune system, acting only to restore skin homeostasis.

In an in vitro study, Khmaladze et al. [197] demonstrated that live *Lactobacillus reuteri* DSM 17938 reduced UVB-induced pro-inflammatory IL-6 and IL-8 production. Interestingly, the authors observed similar anti-inflammatory activity for the corresponding bacterial lysate, indicating that both probiotics and postbiotics may contribute to photoprotection through different mechanisms.

Evidence from animal studies also supports a role for probiotics in photoprotection. In an animal study, Sugimoto et al. [91] showed that application of *Bifidobacterium breve* following UV exposure inhibited the increase in elastase activity and IL-1β levels in the skin.

Overall, both probiotics and postbiotics demonstrate potential for protecting the skin against UV-induced damage. However, it should be emphasized that a considerable proportion of the currently available evidence relates to non-viable microbial preparations rather than true probiotics. Future studies should clearly distinguish between these categories to improve mechanistic understanding and facilitate comparison of clinical outcomes.

## 8. Future Directions

Although both probiotics and postbiotics show promise in protecting the skin, the current body of evidence is considerably stronger for postbiotic preparations and probiotic-derived ingredients than for live probiotic microorganisms. This disparity is largely attributable to the technological challenges associated with incorporating viable microorganisms into topical formulations.

The use of live probiotics in cosmetic products remains particularly challenging because it requires maintaining microbial viability during manufacturing, storage, and application, while simultaneously ensuring compliance with strict microbiological safety requirements. Furthermore, conventional preservative systems used in cosmetic formulations may negatively affect probiotic survival and functionality.

One of the most promising strategies to overcome these limitations is microencapsulation, which enables the stabilization and protection of live microorganisms by enclosing them within protective carrier materials. Such systems may improve bacterial viability, protect probiotics from environmental stresses and preservative exposure, and facilitate their controlled release upon application to the skin. The selection of appropriate encapsulation materials remains crucial, as it directly influences particle stability, bacterial survival, and the effectiveness of delivery.

Consequently, future research should focus not only on elucidating the mechanisms underlying microbiome–skin interactions but also on the development of innovative formulation technologies that enable the safe and effective use of live probiotics in dermatological and cosmetic products. Well-designed clinical studies comparing probiotics, postbiotics, and other microbiome-derived ingredients are also needed to establish their relative efficacy in photoprotection and anti-aging applications.

## 9. Conclusions

The skin microbiome plays a crucial role in maintaining skin homeostasis, barrier integrity, immune regulation, and protection from environmental stressors. Dysbiosis is associated with the development and progression of several inflammatory skin conditions, including atopic dermatitis, psoriasis, and acne vulgaris, underscoring the growing interest in therapeutic strategies targeting the microbiome. Prebiotics, probiotics, and postbiotics represent promising tools for modulating the microbiome in both dermatological and cosmetic applications. Among these approaches, postbiotics and probiotic-derived ingredients currently constitute the majority of available scientific evidence, demonstrating beneficial effects on skin hydration, barrier function, inflammation, oxidative stress, and protection against UV damage. On the other hand, although live probiotics have shown encouraging results in selected experimental and clinical studies, the available evidence remains relatively limited.

All of this highlights the need for further research into the potential of the skin microbiome, with particular emphasis on the role of probiotics, as well as the development of innovative strategies for microbiome modulation and safe, effective dermatological and cosmetic formulations.

## Figures and Tables

**Table 1:** Representative microbiota of major skin microenvironments.

Skin Microenvironment	Major Bacterial Taxa	Major Fungal Taxa
Sebaceous	*Cutibacterium acnes*, *C. granulosum*, *Staphylococcus epidermidis*	*Malassezia restricta*, *M. globosa*
Moist	*Staphylococcus epidermidis*, *Staphylococcus hominis*, *Corynebacterium* spp.	*Malassezia* spp.
Dry	*Cutibacterium* spp., *Corynebacterium* spp., *Betaproteobacteria*, *Flavobacteriales*	*Mixed fungal communities*

**Table 2:** Factors influencing newborn skin colonization.

Factor	Impact on Colonization	Reference
Skin pH	In newborns, the higher skin pH of 6.34–7.5 promotes colonization of the skin surface by pathogenic bacteria.	Fluhr et al. [38] Oranges et al. [39]
Humidity	The skin microbiome of newborns resembles that of moist adult skin. Therefore, moist areas (groin, neck) may be colonized earlier than dry areas.	Oranges et al. [39]
Sebum level	The sebaceous areas are more even and richer due to the influence of maternal hormones, which supports the development of lipophilic bacteria of the *Cutibacterium* genus.	Oranges et al. [39] Capone et al. [40]
Stratum corneum	Still immature, permeable, facilitates the settlement of bacteria, but is better hydrated than in adults.	Oranges et al. [39] Capone et al. [40]

## Data Availability

No new data were created or analyzed in this study. Data sharing is not applicable to this article.
